# Cost-Effectiveness Analysis of a Transparent Antimicrobial Dressing for Managing Central Venous and Arterial Catheters in Intensive Care Units

**DOI:** 10.1371/journal.pone.0130439

**Published:** 2015-06-18

**Authors:** Franck Maunoury, Anastasiia Motrunich, Maria Palka-Santini, Stéphanie F. Bernatchez, Stéphane Ruckly, Jean-François Timsit

**Affiliations:** 1 Statesia, Le Mans, France; 2 3M Company, Neuss, Germany; 3 3M Company, St. Paul, United States of America; 4 Grenoble University Hospital, Grenoble, France; 5 IAME UMR1137-Team 5 Decision Sciences in Infectious Disease Prevention, Control and Care, Paris Diderot University-Inserm, Sorbonne Paris Cité, Paris, France; 6 Paris Diderot University—Bichat University hospital—Medical and Infectious Diseases Intensive care unit, Paris, France; Bambino Gesù Children's Hospital, ITALY

## Abstract

**Objective:**

To model the cost-effectiveness impact of routine use of an antimicrobial chlorhexidine gluconate-containing securement dressing compared to non-antimicrobial transparent dressings for the protection of central vascular lines in intensive care unit patients.

**Design:**

This study uses a novel health economic model to estimate the cost-effectiveness of using the chlorhexidine gluconate dressing versus transparent dressings in a French intensive care unit scenario. The 30-day time non-homogeneous markovian model comprises eight health states. The probabilities of events derive from a multicentre (12 French intensive care units) randomized controlled trial. 1,000 Monte Carlo simulations of 1,000 patients per dressing strategy are used for probabilistic sensitivity analysis and 95% confidence intervals calculations. The outcome is the number of catheter-related bloodstream infections avoided. Costs of intensive care unit stay are based on a recent French multicentre study and the cost-effectiveness criterion is the cost per catheter-related bloodstream infections avoided. The incremental net monetary benefit per patient is also estimated.

**Patients:**

1000 patients per group simulated based on the source randomized controlled trial involving 1,879 adults expected to require intravascular catheterization for 48 hours.

**Intervention:**

Chlorhexidine Gluconate-containing securement dressing compared to non-antimicrobial transparent dressings.

**Results:**

The chlorhexidine gluconate dressing prevents 11.8 infections /1,000 patients (95% confidence interval: [3.85; 19.64]) with a number needed to treat of 85 patients. The mean cost difference per patient of €141 is not statistically significant (95% confidence interval: [€-975; €1,258]). The incremental cost-effectiveness ratio is of €12,046 per catheter-related bloodstream infection prevented, and the incremental net monetary benefit per patient is of €344.88.

**Conclusions:**

According to the base case scenario, the chlorhexidine gluconate dressing is more cost-effective than the reference dressing.

**Trial Registration:**

This model is based on the data from the RCT registered with www.clinicaltrials.gov (NCT01189682).

## Introduction

Catheter-related bloodstream infections (CRBSIs) are associated with attributable mortality rates of up to 11.5% and additional intensive care unit (ICU) length of stay of up to 12 days [1,2]. The broadly accepted method for minimizing CRBSIs is a bundle of care combining maximal sterile barrier precautions for insertion, an appropriate antiseptic solution for skin antisepsis and line access, preferential subclavian catheterization, and immediate removal of unnecessary catheters [3,4]. Combining this catheter-care bundle with continuous quality improvement programs can decrease the CRBSI rate below 2 per 1,000 central venous catheter (CVC)-days [5,6]. In Europe, the incidence of CRBSIs ranges from 1 to 3.1 per 1,000 patient-days [7] and according to the French surveillance network, less than one CRBSI occurred per 1,000 CVC-days in 2010 [8]. However, rates below 2 per 1,000 CVC-days are difficult to achieve in all ICUs [9,10] and in the long term [11].

Most organisms responsible for short-term CRBSIs originate from the insertion site [12]. It has been previously demonstrated that the risk of developing CRBSIs can be dramatically reduced (60% decrease) by the systematic use of a new antimicrobial transparent dressing [13] containing a Chlorhexidine Gluconate (CHG) gel even though bundles of care are appropriately followed and CRBSI level is lower than 1.5 per 1,000 catheter-days in the control group.

The purpose of this work is to evaluate the advantages of the routine use of the new CHG dressing to secure central lines of patients in ICU from a medico-economic viewpoint compared to non-antimicrobial transparent dressings, in settings where bundles of care practices are appropriately followed and where incidence of infection is already low (below 2 per 1,000 catheter-days [13,14]). Both medical and economic criteria are embedded into a decision-analytic model to support the choice of the best dressing strategy from an ICU perspective.

## Methods

### Study Design

The adopted modeling approach complies with the guidelines of French National Authority for Health (Haute Autorité de Santé—HAS) [15]. The 30-day ICU-time non-homogeneous Markov model [16–18] structure was based on observed data of a multicentre randomized controlled trial (RCT) [13], conducted by the Grenoble University Hospital—CHU Grenoble (ClinicalTrials.gov Identifier: NCT01189682). The model has been programmed using Visual Basic Application with the Excel 2007 software.

### Data Collection

The main data source was the database assembling all patient data collected during the RCT [13]. This multicentre randomized-controlled study compared the impact of the antimicrobial 3M Tegaderm CHG (referred in the current study as CHG dressings) and of non-antimicrobial transparent dressings (referred as non-CHG dressings) on the rate of catheter related infections. The main objective of the RCT transposed in this cost-effectiveness analysis was to determine if the use of the new transparent CHG dressing decreased CRBSI rates. The RCT was not blinded to the investigators or ICU staff due to the obvious visual differences between the dressings, but was blinded to the microbiologists processing the skin and catheter cultures and to the committee adjudicating on the CRBSI cases. The two groups receiving different types of non-antimicrobial transparent dressings in the RCT were pooled together as “non-antimicrobial transparent dressings” for the purpose of the modeling presented in this paper.

### Study Population

The multicentre RCT [13] enrolled adult patients (>18 years) admitted to 12 French ICUs in seven universities and four general hospitals, from 31 May 2010 to 29 July 2011, and expected to require intravascular catheterization for 48 hours. Patients with known allergies to chlorhexidine or transparent dressings were excluded. Of 2,054 screened patients with at least one catheter, 1,898 could be enrolled in the study and 1,879 were assessable for the intention-to-treat analysis, for a total of 4,163 catheters and 34,339 catheter-days. Patients and catheters characteristics are reported in the Results section.

### Study Catheters

In the RCT [13], all central venous catheters inserted at subclavian, jugular and femoral veins, as well as arterial catheters inserted at radial and femoral arteries for a given patient, were managed according to the randomized dressing assignment. Pulmonary arterial, hemodialysis, and peripherally-inserted venous catheters and catheters inserted before ICU admission were excluded from the study. All study centers followed French recommendations for catheter insertion and care, which are similar to Center for Disease Control (CDC) recommendations [19].

### Endpoints

The final health outcome of the cost-effectiveness analysis is the number of CRBSIs avoided and the cost-effectiveness criterion is the cost per patient with CRBSI avoided resulting from chlorhexidine dressing use.

### Modeling and Statistical Analysis

Markov models simulate the trajectory of patients among distinct states of health over time [20–23]. The main assumption of state-transition Markov models is that the next health state depends only on the present state and not on the sequence of events that preceded it. Eight health states were considered (Table 1), four combining either occurrence, or no occurrence, of CRBSI, and the need, or no need, of a new central line (CT); one for contact dermatitis; one for changing to an alternative dressing (gauze and tape) in case of dermatitis, and two absorbing states (death and discharge from the ICU).

**Table 1:** Health states defined from a multicentre randomized controlled trial [13].

Health States	Definition
1. No CRBSI / No new CT needed	Insertion of a first catheter, no diagnosed CRBSI and no contact dermatitis
2. No CRBSI / new CT needed*	No diagnosed CRBSI, no contact dermatitis and a new catheter inserted (not as a replacement)
3. CRBSI / No new CT needed	CRBSI diagnosed without neither contact dermatitis nor the need for inserting a new catheter
4. CRBSIs / new CT needed*	CRBSI diagnosed without contact dermatitis but the need for inserting a new catheter
5. Contact dermatitis	No diagnosed CRBSI, and no need for new catheter inserted but occurrence of contact dermatitis
6. Dressing Gauze and Tape	Change to an alternative dressing strategy (gauze and tape)
7. Discharge	Patient leaves the ICU alive
8. Death	Patient dies during the ICU stay

* New CT needed can mean either the replacement of the existing catheter, or the need for an additional catheter at a new site.

CRBSI, Catheter-related Bloodstream Infections; CT, Catheter (Central venous or radial / femoral arterial).

**Fig 1:**
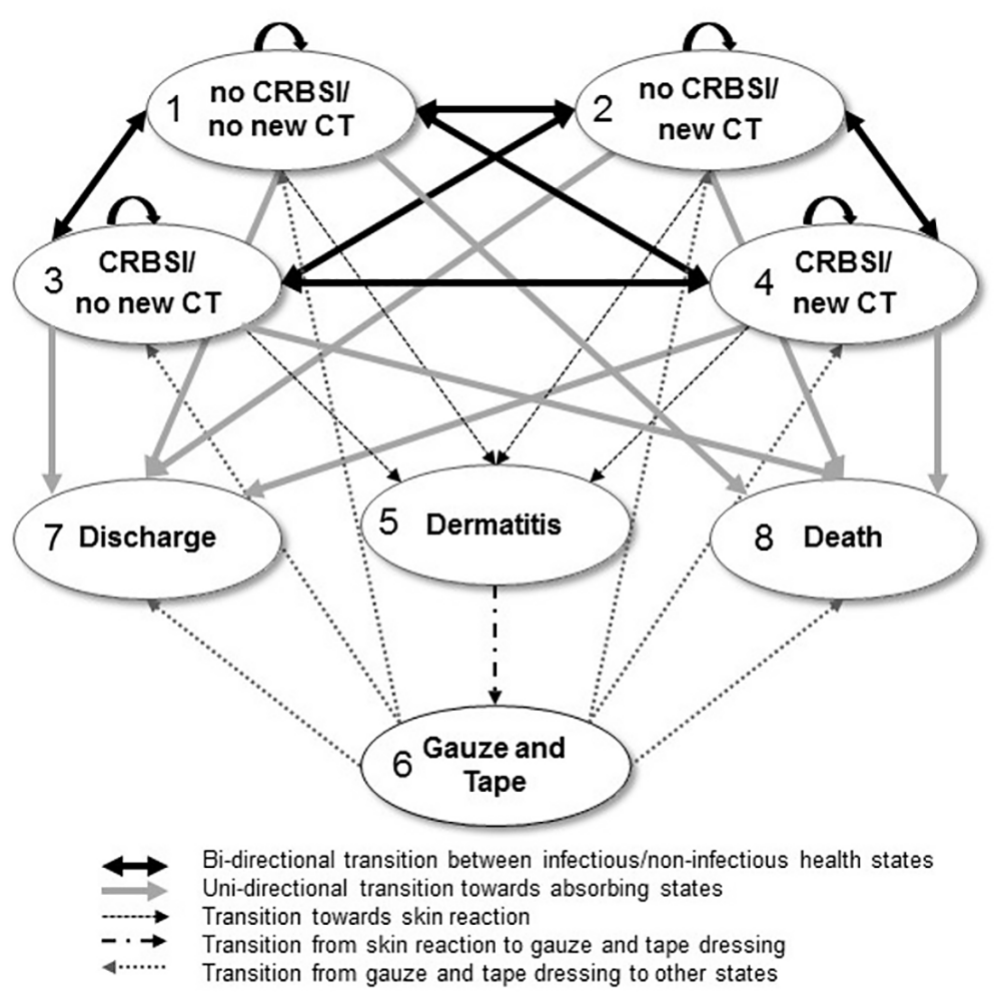
Structure of the Markov Model showing the possible transition between health states from one Markov cycle to the next cycle. The costs per patient for each health state were calculated in both CHG and No-CHG dressing as respectively: State 1: €1,270 and €1,266; State 2: €1,364 and €1,361; State 3: €13,661 and €13,658; State 4: €13,756 and €13,752; State 5: €1,388 and €1,385; State 6: €1,266 and €1,266; State 7: €0 for both groups; State 8: €0 for both groups; CHG: chlorhexidine gluconate; CRBSI: catheter-related bloodstream infection; CT: catheter.

The statistical unit of the study is the ICU patient within a time horizon of 30 days (including patients discharged alive from the ICU, alive but still in the ICU, or deceased during the ICU stay). Patient data from the multicentre RCT (source study) [13], comparing the 3M Tegaderm Chlorhexidine Gluconate (CHG) Securement dressing to non-antimicrobial transparent dressings, were translated into a daily patient transition matrix among the different possible health states, for both the antimicrobial and non-antimicrobial dressing groups (see transition matrices in S1 and S2 Tables in Supporting Information). Data was censored beyond 30 days. The transition matrixes were used to perform non-homogeneous Markov-Chain Monte Carlo (NH-MCMC) simulations [24] representing the observed daily evolution of patients in ICU. The possible transitions among health states from one day to the next are represented in the Markov diagram (Fig 1). The Markov cycle duration corresponds to one day. One thousand Monte Carlo simulations of 1,000 patients were used for probabilistic sensitivity analysis and 95% confidence intervals (CI) calculations.

### Main Assumptions

1. In the cases where the "Discharge" state was reported, and a CRBSI was observed for this patient up to two days after the event, the infection was considered to occur the day of discharge from the ICU.
2. The transitional probability from the health state "Contact Dermatitis" to “Dressing Gauze and Tape” state was considered the same for both groups. By entering to the “Dressing Gauze and Tape” the patient followed probabilities of transition corresponding to the non-CHG dressings arm.
3. The cost of CRBSI is independent from the outcome (survival or death or discharge).
4. Catheter colonization with or without CRBSIs was considered as having negligible diagnosis costs and was excluded from the model for not being considered as a “health state” *per se*.
5. The costs related to replacement of a catheter suspected to be colonized (and causing CRBSI) were comprised in one of the health states including the need for a new central line. The cost per ICU day was considered as identical for each dressing group. The cost of a gauze and tape dressing is identical in both groups.
6. Health states including CRBSIs were assumed to last a single day because it was not technically possible to identify the termination of a CRBSI in the patient database. However, the costs of treating the complete episode, as well as the total costs associated with the extra length of stay due to the CRBSI were accounted on the day when the CRBSI was diagnosed.

#### Base case input parameters considered in the cost analysis

The base case analysis is the most representative case of the real life, considering French ICU settings, and depending on expert opinions, literature, and RCTs.

The main input parameters considered in the cost analysis are the following, in €2013:

- Dressing costs per day: CHG dressing, €3.59 [13]; non-antimicrobial transparent film, €0.18; gauze and tape, €0.06.
- Cost of treating contact dermatitis (mean/episode): catheter removal, €23.62 [25]; four gauze and tape dressings, €0.24; catheter insertion, €94.87. Note that the skin lesions themselves healed spontaneously upon removal of the transparent dressings, without further negative health impact or treatment costs.
- Direct cost of treating CRBSI (mean/episode) [25]: €580.26.
- Cost per ICU [26]: €1,265.93 per day.
- Additional ICU Length of stay (LOS) due to CRBSI: 9.33 days (NH-MCMC calculation).
- Cost of added ICU LOS due to CRBSI: €11,811.13 (NH-MCMC calculation).
- Cost per catheter change (venous + arterial: 50%-50%) [25]: €94.97.
- Overall cost of one CRBSI (direct cost of treating one CRBSI plus cost of additional ICU LOS due to CRBSI): €12,391.40 (calculation).

Direct costs for the treatment of CRBSIs were obtained from a micro-costing study [25]. ICU costs were based on an observational (real life) study [26] that assessed all resources consumed during a patient day in the ICU. This twenty-four hours multicentre prospective medico-economic study provides a complete overview and estimation of the actual average cost for medical and surgical ICUs in different hospital types in France: Hospitals (CH), University Hospitals (CHU) and Regional Hospitals (CHR). Twenty-two ICUs were selected randomly and all costs for 109 patients were estimated. For patients with CRBSI, an additional cost [27] due to an extra ICU length of stay (LOS) was calculated (see next section).

#### Additional ICU LOS due to CRBSIs and comparability of patients’ subgroups with or without CRBSIs

In order to assess the impact of CRBSIs on extending ICU LOS, a subgroup analysis was performed comparing patients having developed a CRBSI during the ICU stay with those who did not have a CRBSI. The comparison was made through independent non-homogeneous MCMC simulations for each dressing group (CHG and Non-CHG).

The non-homogeneous Markov Chain Monte Carlo simulation in each group estimated additional ICU LOS due to CRBSIs as of 8.55 days and 10.1 days for the CHG and Non-CHG strategies, respectively. For the base case scenario, we set an extra ICU LOS of 9.33 days, which was an average between the two strategies.

#### Costs per Markov state per patient

The calculation of the cost for each Markov state per patient was done as follows (using the base case input parameters listed above):

- Dressing costs (including time needed per dressing, number of nurses involved, and materials used [25]) and cost per ICU day [26] were taken into account for health states 1–6;
- Cost of treating contact dermatitis [25]–(including catheter removal, four alternative dressings, and insertion of a new catheter) was taken into account only for health state 5;
- Cost of treatment of CRBSI [25] and additional ICU-LOS due to CRBSI [13,25] were taken into account for health states 3 and 4;
- Cost per catheter change (venous, arterial) [25] was taken into account for health states 2 and 4.

#### Adjustments on covariates between the subgroups

A statistical analysis for all confounding covariates, such as age, sex, Sequential Organ Failure Assessment severity score (SOFA, a score predicting ICU mortality based on lab results and clinical data [28]), duration of catheterization, number of dressing change per day, was performed in order to demonstrate the comparability between the subgroups. Four subgroups of patients (CHG/CRBSI, CHG/No-CRBSI, Non-CHG/CRBSI, Non-CHG/No-CRBSI) were compared with these covariates. Mann-Whitney tests between subgroups were performed.

#### Sensitivity analyses

Sensitivity analyses are performed to vary each parameter of the model in order to determine what levels will result in a change of preference for the therapeutic strategy. This is a way to test the boundaries of the model and identify the main parameters driving cost differences.

One-way sensitivity analyses were performed varying the main input parameters (additional ICU LOS due to CRBSI (days), 3M Tegaderm CHG Dressing cost (€2013), number of CHG dressing per day, number of Non-CHG dressing per day, and cost per ICU day) of the model around the base case assumptions.

A probabilistic sensitivity analysis [29] was performed with 1,000 non-homogeneous MCMC simulations of 1,000 patients for both CHG dressing and non-CHG dressing groups. Each group of 1,000 patients depicts an average patient representing all patients for each dressing group studied in the RCT [13] The method used was the Gibbs sampling [30], a commonly used Markov Chain Monte Carlo algorithm. It allowed to retrace 10^6^ health trajectories (1,000 x 1,000 patients for each dressing strategy), based on the probabilities observed in the RCT [13] day-after-day (during 30 days) for each patient to change from one health-state to another. Repeating the algorithm 1,000 times allows the calculation of 95% confidence intervals for the cost-effectiveness criterion (here, number of CRBSI avoided and cost per patient). The health states including CRBSI (CRBSI/No new catheter and CRBSI/new catheter) as rare events for both strategies are in the area of low probabilities. On the other hand, the “discharge” and “death” states as frequent events for both strategies are in the area of high probabilities. This corresponds to the reality observed in the RCT (higher frequency of discharge and death than CRBSI).

## Results

### Impact of the covariates: Patients and Catheters Characteristics

The 4 subgroups of patients (CHG/CRBSI, CHG/No-CRBSI, Non-CHG/CRBSI, Non-CHG/No-CRBSI) were similar in regards to the SOFA score, age, sex, exposure to the risk factor “duration of catheterization” and daily number of dressing(s) needed (Table 2). The results of Mann-Whitney tests on these covariates between subgroups (CRBSI/No CRBSI) show no statistically significant difference at the 0.05 level.

**Table 2:** Comparability of subgroups on covariates.

Dressing group	CHG * Mean (std)	Non-CHG ** Mean (std)	Comparison p-value ⱡ
SOFA score (severity)			
CRBSI	7.89 (4.08)	10.29 (3.39)	0.1459
No CRBSI	8.17 (3.76)	8.17 (3.83)	0.8737
Age (years)			
CRBSI	58.78 (13.73)	62.57 (19.08)	0.5262
No CRBSI	61.97 (15.71)	62.17 (16.42)	0.6043
Number of males			
CRBSI	5 (55.56%)	12 (57.14%)	1.0000
No CRBSI	630 (68.11%)	603 (65.97%)	0.3460
Catheterization time (days)			
CRBSI	39.67 (22.58)	28.43 (31.56)	0.0984
No CRBSI	11.01 (11.52)	10.92 (11.01)	0.9934
Number of dressings per day			
CRBSI	0.59 (0.29)	0.73 (0.37)	0.2675
No CRBSI	0.67 (0.52)	0.65 (0.58)	0.2653

* CHG group frequencies: 9 patients with CRBSI, 925 patients without CRBSI.

** Non-CHG group frequencies: 21 patients with CRBSI, 914 patients without CRBSI.

^ⱡ^ The results (p value) of Mann-Whitney tests on these covariates between subgroups show no statistically significant difference if p>0.05 (at a 0.05 level).

CHG, chlorhexidine gluconate; SOFA, Sequential Organ Failure Assessment; CRBSI, catheter-related bloodstream infection.

### Cost-Effectiveness Analysis

A ratio of 1 to 5 was observed for the average number of CRBSIs between CHG and non-CHG dressing groups. CRBSI occurred for 3 and 14 patients in each CHG and non-CHG groups respectively (1,000 patients in each group; Table 3). This difference was highly statistically significant as indicated by the non-overlapping 95% confidence intervals (CI). The number of ICU-days as well as the number of days before discharge and death occurred was comparable in the two groups. Moreover, the number of patients entering both absorbing states, coded as 7 (discharge from ICU) and 8 (death), was comparable in both groups of dressings.

**Table 3:** Occurrences per 1,000 patients as generated through 1,000 NH-MCMC of 1,000 patients in each dressing group, according to the base case scenario.

Study arm	CHG dressing			Non-CHG dressing		
Statistics	Mean (%ₒ)	Lower 95%CI	Upper 95%CI	Mean (%ₒ)	Lower 95%CI	Upper 95%CI
State 1 no CRBSI / no new CT (at the beginning of the simulation)	1,000	1,000	1,000	1,000	1,000	1,000
State 2 no CRBSI / new CT	278.2	241.8	314.5	251.6	218.8	284.4
State 3 CRBSI / no new CT	0.00	0.00	0.00	5.3	0.7	9.8
State 4 CRBSI / new CT	3.1	0.00	64.8	9.5	3.3	15.7
State 5 Contact Dermatitis	28.8	14.6	43.0	12.7	4.4	20.9
State 6 G+T dressing	28.8	14.6	43.0	12.7	4.4	20.9
State 7 (ICU Discharge)	604.1	574.1	633.8	613.4	582.8	644.1
State 8 (Death)	263.7	234.7	292.7	270.7	242.4	299.0
Number of ICU-days	12.91	12.30	13.52	12.72	12.12	13.32
Number of days before State 7 Discharge	18.74	18.05	19.43	18.43	17.72	19.16
Number of days before State 8 Death	25.17	24.49	25.85	25.28	24.64	25.92

CHG, Chlorhexidine Gluconate; CI, Confidence Interval; CRBSI, Catheter-related bloodstream infection; CT, Catheter; ICU, Intensive Care Unit; NH-MCMC, Non-Homogeneous Markov Chain Monte Carlo.

For a 30-day time horizon in ICU, the mean cost per patient for CHG group was of €16,461, versus €16,320 for the non-CHG strategy. The mean cost per patient with CRBSI was of €39,071 and €41,424 in CHG and non-CHG dressing groups while the mean cost per patient without CRBSI was of €16,385 and €15,946 in CHG and non-CHG dressing groups, respectively (Table 4). Subgroup analyses supported by the comparability test (Table 2) compared the average total costs for patients with CRBSI versus patients without CRBSI for each study group (CHG and Non-CHG dressings). This comparison revealed no significant differences in costs among the subgroups (Table 4).

**Table 4:** Mean Cost for one patient in each dressing group.

Groups /Statistics	Mean	Lower 95%CI	Upper 95%CI
ALL PATIENTS			
CHG (1)	€16,461	€15,659	€17,265
Non-CHG (2)	€16,	€15,538	€17,103
Diff. Cost (1–2)	€141	€-975	€1,258
PATIENTS with CRBSI in ICU			
CHG (1)	€39,071	€17,384	€60,758
Non-CHG (2)	€41,424	€36,213	€46,635
Diff. Cost (1–2)	€-2,353	€-24,984	€20,277
PATIENTS without CRBSI			
CHG (1)	€16,385	€15,584	€17,186
Non-CHG (2)	€15,946	€15,177	€16,715
Diff. Cost (1–2)	€439	€-664	€1,542

Time Horizon: 30-days ICU—1,000 NH-MCMC simulations of 1,000 patients (€2013).

CHG: Chlorhexidine Gluconate; CI: Confidence Interval; ICU: Intensive Care Unit; NH-MCMC: Non-Homogeneous Markov-Chain Monte Carlo simulation

### One-way Sensitivity Analysis

**Fig 2:**
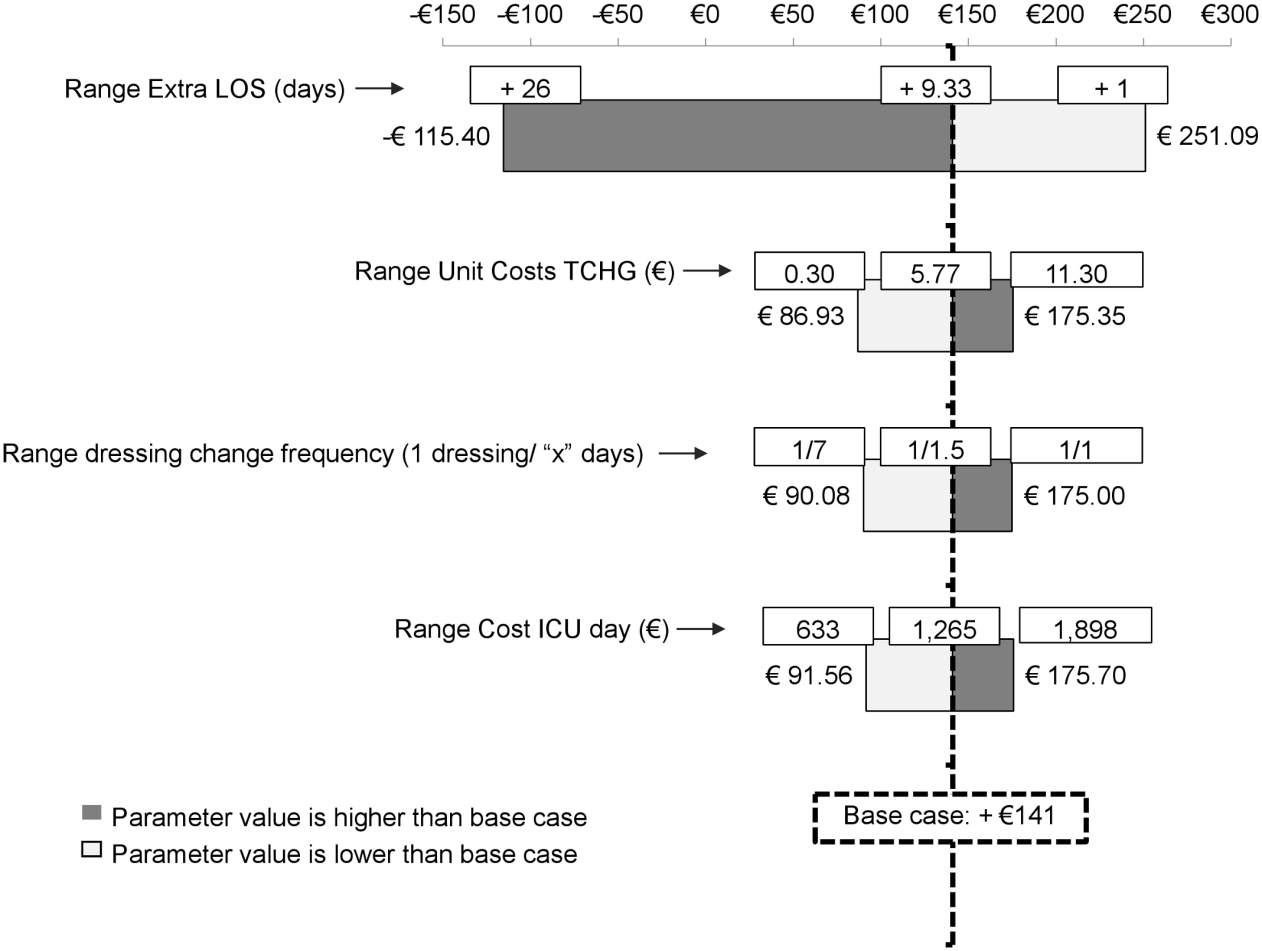
Tornado diagram for the One-way Sensitivity Analysis. This diagram illustrates the impact of the variation in some parameters of the model on the cost difference between the strategies. The base case is average cost difference (€+141) between the two dressing strategies for the parameter’s values indicated on the “y” axis. The tested range for each parameter is indicated by the arrows. The main driver parameter for cost difference is the Extra LOS associated to CRBSI. ICU: Intensive Care Unit; CRBSI: Catheter-related bloodstream infection; CHG: Chlorhexidine Gluconate; LOS: Length of Stay.

A tornado diagram (Fig 2) shows the variation in the mean cost difference between the CHG and non-CHG strategies around the one calculated for the base-case (€141). The model was most sensitive to the variation of the number of extra ICU LOS due to CRBSIs. The cost difference varied of approximately €370, when accounting from a single extra ICU day (cost difference of €251) to 26 extra ICU days (cost difference of €-115). The next three influential parameters were the CHG-dressing cost, the interval for dressing change, and the cost per ICU-day. However, the variation in the cost differences obtained by changing these parameters was less pronounced (differences between upper and lower limits of €88, €85 and €83, respectively).

### Probabilistic Sensitivity Analysis (PSA)

**Fig 3:**
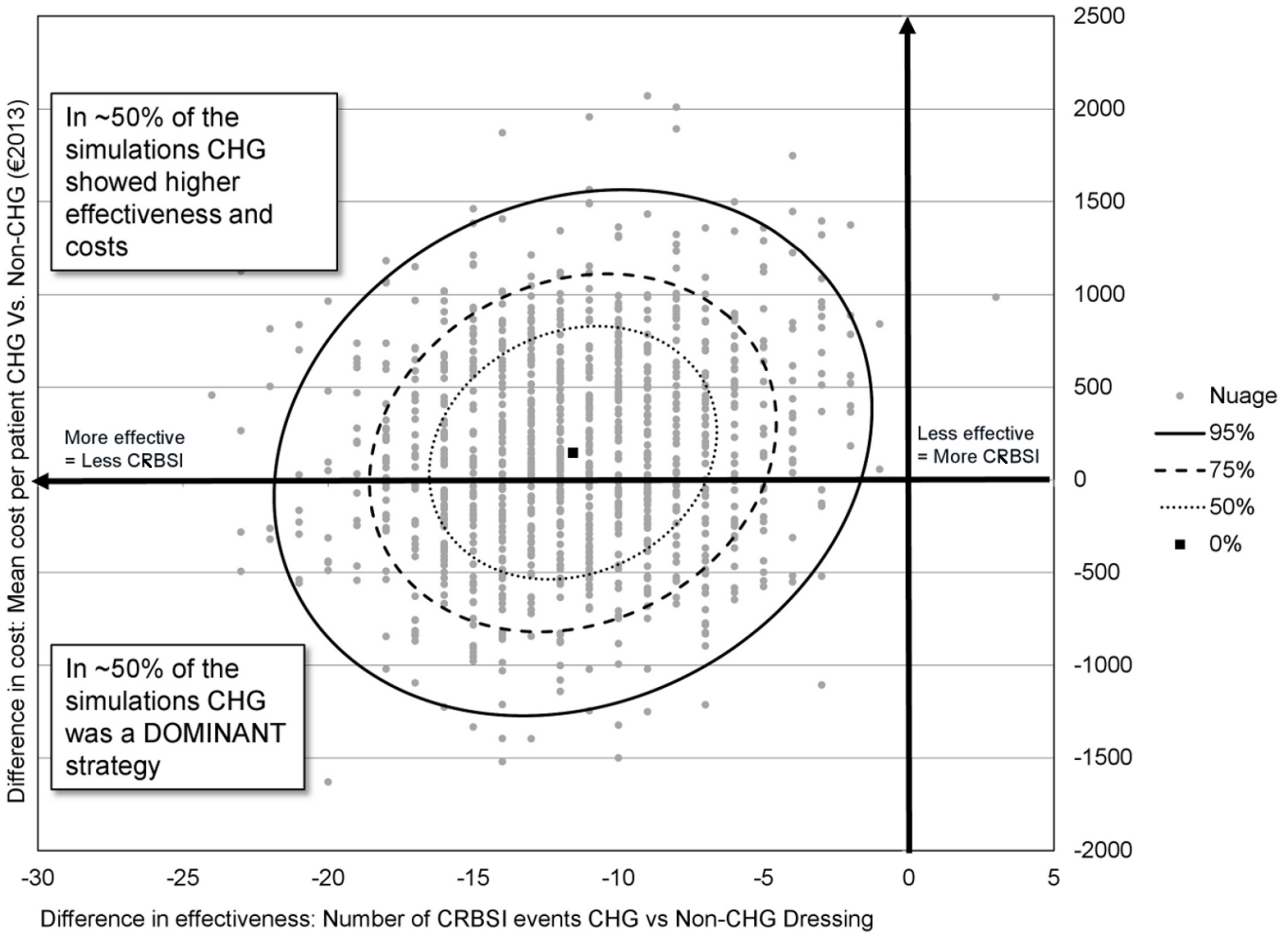
Cost-effectiveness results for the probabilistic sensitivity analysis. The analysis uses 1,000 non-homogeneous Markov-Chain Monte Carlo simulations of 1,000 patients for each dressing strategy. The x axis represents the difference in effectiveness (number of CRBSI events in CHG versus non CHG dressing) and the y axis represents the difference in cost (mean cost per patient with CHG versus non CHG dressing) in €2013. The (0,0)-point indicates the reference dressing strategy (Non-CHG group). Each point in the graph represents the Incremental Cost-Effectiveness Ratio (ICER) of CHG-dressing strategy versus reference dressing. All but three points are at the left side of the graph, showing that CHG dressing strategy was 99.7% more effective than the comparator at the same costs per patient. The squared point in the center of the cloud represents the average CE ratio of all 1,000 simulations. CHG: chlorhexidine gluconate; CRBSI: catheter-related bloodstream infection.

The PSA cost-effectiveness plan (Fig 3) describes the difference in the effectiveness on the x-axis and the difference in cost on the y-axis between the two groups of dressings, for 1,000 NH-MCMC simulations of 1,000 patients in each group. The (0,0)-point indicates the reference dressing strategy (Non-CHG group). All other points observed on the graph represent the incremental cost-effectiveness ratio (ICER: Difference in costs / Difference in effectiveness [15]) of CHG-dressing strategy versus reference dressing. This PSA supports the decision to adopt the CHG dressing for critically ill patients since the strategy is 99.7% more effective than the comparator at the same cost per patient in the intensive care unit (only 3 points over 1,000 were observed in the cost-effectiveness plan where the assessed product was less effective than the reference dressing). The incremental cost-effectiveness ratio (ICER) is of €12,046 per CRBSI prevented, which is far less than the cost of caring for an infected patient in the ICU setting, estimated here to be around €40,000 (Table 4).

The average incremental net monetary benefit (iNMB) per patient induced by using CHG- instead of non-CHG dressings in ICU can be calculated by valuing the net health gain in monetary terms, based on the current willingness to pay (WTP) minus the difference in cost per patient between each compared strategy, as follows:

iNMB = difference in effectiveness per patient x WTP – difference in cost per patient.

In the current context, the willingness to pay (WTP) was considered as the mean cost for treating one patient with CRBSI included in the reference dressing arm. The mean “incremental net monetary benefit” obtained was positive (€344 (95%CI: [€-883; €1,573])), indicating that the assessed technology is cost-effective.

## Discussion

Several studies highlighted the clinical and economic impact of catheter-related infections [31, 32]. Others pointed out the medical and economic benefits of using CHG dressings [33, 34] or antiseptic impregnated central venous catheters for preventing these infections [35, 36]. Crawford *et al*. [33] estimated that the potential annual U.S. net benefits from using chlorhexidine sponge dressing ranged from $275 million to approximately $1.97 billion. According to Ye *et al*. [34] the systematic use of chlorhexidine gluconate (CHG)-impregnated sponge dressing could avoid 35 CRBSIs, 145 local infections, and 281 intensive care unit days and save about $895,000 annually in a hypothetical 400-bed hospital inserting 3,078 central venous catheters (CVCs) per year. Schwebel *et al*. [25] showed that the expected savings per catheter when using CHG dressings were of US $117 with a 3-day dressing change schedule and US $98 with a 7-day dressing change schedule. In the current study, we estimated that CHG dressing prevents 11.74 infections per 1,000 patients via probabilistic cost-effectiveness sensitivity analysis. Through the systematic use of the CHG dressing, the adjusted cost per patient was on average €16,461, the cost difference per patient was of €141, and incremental net monetary benefit per patient of €344. Beyond the “cost saving” aspects pointed out by authors cited above, our analysis demonstrates that the use of CHG dressings in ICUs is cost-effective as indicated by a positive iNMB [37,38].

The one-way sensitivity analysis identified the co-variables impacting most the cost-effectiveness calculations. Additional ICU LOS due to CRBSIs appears as the main driver of the model. The next more important variables are the CHG dressing unit price, the number of dressings per day and the cost per ICU day. These results are consistent with the health-economics analysis for a CHG sponge dressing published by Schwebel *et al*. [25].

The non-homogeneous Markov Chain Monte Carlo (NH-MCMC) simulation represents an innovative analytical approach for modeling healthcare-acquired infections. The literature in this field offers only examples based on static decision tree models, used for both cost-effectiveness or cost-benefit studies [25,33,34]. The more remarkable feature of the current NH-MCMC simulation relates to the fact that it is based on daily real-life raw data, and not on published mean values found in the literature. The time-dependence addressed here (i.e. evolution of the risk of developing a CRBSI with increased catheterization time) corroborates that the “micro” simulation approach chosen is suitable considering the nature of the available data (daily observations).

This model has some limitations. First, it was built on a single clinical study because it was the only RCT available with this particular product. Second, the cost-effectiveness analysis was based on a scenario specific to French ICUs, where the CRBSI rates are rather low (below 2 per 1,000 catheter-days [13,14]). As a consequence, the NH-MCMC model cannot be directly transposed to other settings or other countries with different CRBSI baseline rates. This transposition would require local individual data on time-dependent probabilities of transition among health states at the daily level, which are not available in general. Further studies involving other countries are needed to generalize our results and therefore our findings do not necessarily predict similar cost effectiveness of CHG dressings in other countries or in specific patients’ subgroups.

Third, the NH-MCMC model very likely underestimated the costs for the non-antimicrobial dressing group, where the number of occurrences of discharge and death (absorbing states with associated null cost) was higher, with 9 discharged patients and 7 deceased patients. As a consequence, the calculated average cost per patient with CHG dressings increased and the corresponding effectiveness decreased. This third limitation is on the positive side since it ensures a conservative cost-effectiveness approach, as recommended by the HAS [15] for the base case analysis. A subgroup analysis based on living patients only, not discharged from the ICU within the specified time horizon, was not possible since there were no CRBSI events in this sub-population.

International guidelines for prevention of catheter-related infections were followed in all study centers participating in the source RCT and the rate of infection was low also in the control group. Furthermore, there was no difference between treatment groups in the covariates (see Table 2). Some studies have shown an increase in infection rate for the femoral insertion site, but this was not observed in our source study (see electronic supplement in [13]).

According to the probabilistic sensitivity analysis, which addresses the level of uncertainty of the results, the CHG-dressing strategy passed the test for cost-effectiveness even in the conservative scenario of very low CRBSI incidence and frequent dressing changes. The transparent antimicrobial dressing is significantly more efficacious to prevent CRBSIs when compared to the reference dressing without any additional cost for the ICU.

This study also has the non-technical limitation of being sponsored by industry (the 3M Company). However, an external research organization (Statesia) was hired to handle independently the development of the simulation model and the data analysis to remove any possible bias. Two employees of the 3M Company worked alongside with non-3M authors for the preparation of the manuscript, with the final version being approved by all non-3M authors prior to submission.

## Supporting Information

S1 Table Transition Matrix for CHG dressings. AE, Adverse Event; CRBSI, Catheter-related bloodstream infections; CT, Catheter; G+T, Gauze and Tape; ICU, Intensive Care Unit.
(XLSX)

S2 Table Transition Matrix for non-CHG dressings. AE, Adverse Event; CRBSI, Catheter-related bloodstream infections; CT, Catheter; G+T, Gauze and Tape; ICU, Intensive Care Unit.
(XLSX)
